# Different states of synaptotagmin regulate evoked versus spontaneous release

**DOI:** 10.1038/ncomms10971

**Published:** 2016-03-22

**Authors:** Hua Bai, Renhao Xue, Huan Bao, Leili Zhang, Arun Yethiraj, Qiang Cui, Edwin R. Chapman

**Affiliations:** 1 Howard Hughes Medical Institute and Department of Neuroscience, University of Wisconsin, Madison, Wisconsin 53705, USA; 2 Department of Chemistry and Theoretical Chemistry Institute, University of Wisconsin, Madison, Wisconsin 53706, USA

## Abstract

The tandem C2-domains of synaptotagmin 1 (syt) function as Ca^2+^-binding modules that trigger exocytosis; in the absence of Ca^2+^, syt inhibits spontaneous release. Here, we used proline linkers to constrain and alter the relative orientation of these C2-domains. Short poly-proline helices have a period of three, so large changes in the relative disposition of the C2-domains result from changing the length of the poly-proline linker by a single residue. The length of the linker was varied one residue at a time, revealing a periodicity of three for the ability of the linker mutants to interact with anionic phospholipids and drive evoked synaptic transmission; syt efficiently drove exocytosis when its tandem C2-domains pointed in the same direction. Analysis of spontaneous release revealed a reciprocal relationship between the activation and clamping activities of the linker mutants. Hence, different structural states of syt underlie the control of distinct forms of synaptic transmission.

## Abstract (web summary)

Synaptotagmin contains tandem Ca^2+^-binding C2-domains that interact with target membranes to trigger exocytosis. Here, Bai *et al*. manipulate the relative orientation of these two domains using a synthetic polyproline linker and show that a parallel orientation is most effective for membrane interaction and exocytosis.

## Graphical abstract

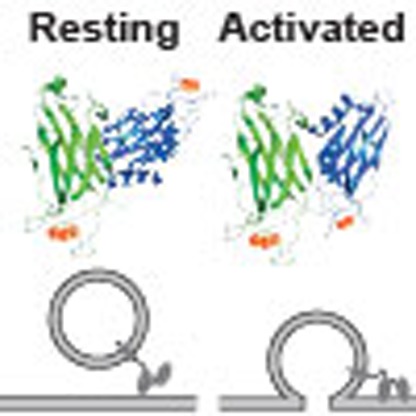

Evoked neurotransmitter release mediates rapid communication between neurons, and thus underlies neural network function^1^. Spontaneous neurotransmitter release also occurs, and this mode of secretion affects myriad aspects of synaptic function including post-synaptic protein synthesis and the maintenance of synaptic contacts^2^,^3^. Synaptotagmin 1 (syt) is an abundant integral membrane protein that is targeted to synaptic vesicles (SVs) where it controls both of these forms of exocytosis, by serving as a Ca^2+^ sensor that triggers evoked release, and by acting as a fusion clamp that prevents exocytosis under resting conditions^4^,^5^,^6^,^7^. It is not known how syt can switch states from an inhibitor to an activator of secretion. This is a puzzling problem, as syt senses Ca^2+^ via tandem C2-domains, C2A and C2B, which comprise most of the cytoplasmic domain of the protein, and these isolated domains do not exhibit significant conformational changes upon binding metal^8^,^9^.

Syt interacts with soluble *N*-ethylmaleimide-sensitive factor (NSF) attachment protein receptors (SNAREs)^10^, which form complexes that are both necessary and sufficient for membrane fusion^11^. In neurons, the SNARE complex consists of the vesicular (v-) SNARE, synaptobrevin (syb), and the target membrane (t-) SNAREs, syntaxin 1 (syx) and SNAP-25. The assembly of v- and t-SNAREs into SNARE complexes provides sufficient energy to drive slow rates of membrane fusion *in vitro*^12^. Syt binds directly to t-SNAREs, but not to v-SNAREs, and inclusion of syt in reconstituted fusion assays renders SNARE catalysed fusion reactions dependent on calcium ions^13^. Additional biochemical studies revealed that Ca^2+^·syt directly regulates the assembly of SNARE complexes^14^,^15^,^16^. However, cell-based experiments provide evidence both for and against the idea that syt·t-SNARE interactions trigger fusion, and a consensus has not been reached^4^,^17^,^18^,^19^,^20^.

Syt also directly interacts with membranes that harbour anionic phospholipids^21^. Upon binding Ca^2+^, the distal tips of Ca^2+^-binding loops 1 and 3 (see Fig. 1 for details) in both C2A and C2B partially insert into the target membrane^4^,^22^,^23^,^24^, potentially generating localized curvature^25^,^26^ and/or pulling the two membranes together to mediate close apposition^15^,^23^,^27^, thereby facilitating fusion. Both *in vitro* and cell-based experiments indicate that Ca^2+^·syt·membrane interactions constitute a crucial step in excitation–secretion coupling^4^,^28^.

The tandem C2-domains of syt are connected by a nine-residue linker that is flexible, and might allow the C2-domains to adopt multiple distinct relative orientations^29^,^30^,^31^,^32^. The objective of the current study was to constrain these C2-domains to begin to determine whether changes in their relative configuration underlie the ability of syt to change functional states, from a clamp to an activator of fusion. This question was addressed by exploiting the properties of poly-proline segments. Proline is a unique amino acid; its side-chain merges with its backbone, thus restricting the backbone dihedral angles to a limited range^33^,^34^. These properties underlie the finding that short poly-proline segments form rigid helices with a periodicity of three (note: the structures of longer poly-proline motifs, beyond 12–15 residues, can be complex because of the increasing probability of introducing *cis* proline conformations^35^). Because of this rigidity, short poly-proline rods have been used as spectroscopic rulers, or rigid spacers, for decades^34^,^35^,^36^,^37^. However, the periodicity of these helices has not been extensively exploited to address protein function and engineering problems.

Here, we replaced the native nine-residue linker of syt with a nine-residue poly-proline rod, and confirmed an earlier study demonstrating that this mutant is functional in terms of driving fusion in both *in vitro* and cell-based assays^4^. Interestingly, molecular dynamics (MD) simulations predicted that the C2-domains in this mutant form of syt were not only highly constrained, but also have a strong tendency to point in the same direction, likely towards the plasma membrane. We then varied the length of the linker, one residue at a time, to systematically alter the relative angle between the tandem C2-domains, and observed a clear periodicity of three regarding the activity of these mutants in a variety of biochemical and functional assays. These observations, in conjunction with the results from photoinduced electron transfer (PET) quenching experiments, demonstrate that the C2-domains were in fact constrained to specific, discernable, orientations. Importantly, there was a clear reciprocal relationship between the abilities of the linker mutants to clamp spontaneous release and to drive evoked release: mutants that drove efficient evoked release failed to inhibit spontaneous release and visa-versa. Hence, syt switches states, from inhibitor to activator of fusion, via dynamic alterations in the relative orientation of its tandem C2-domains.

## Results

### Computational modelling of poly-proline linker mutants

**Figure 1:**
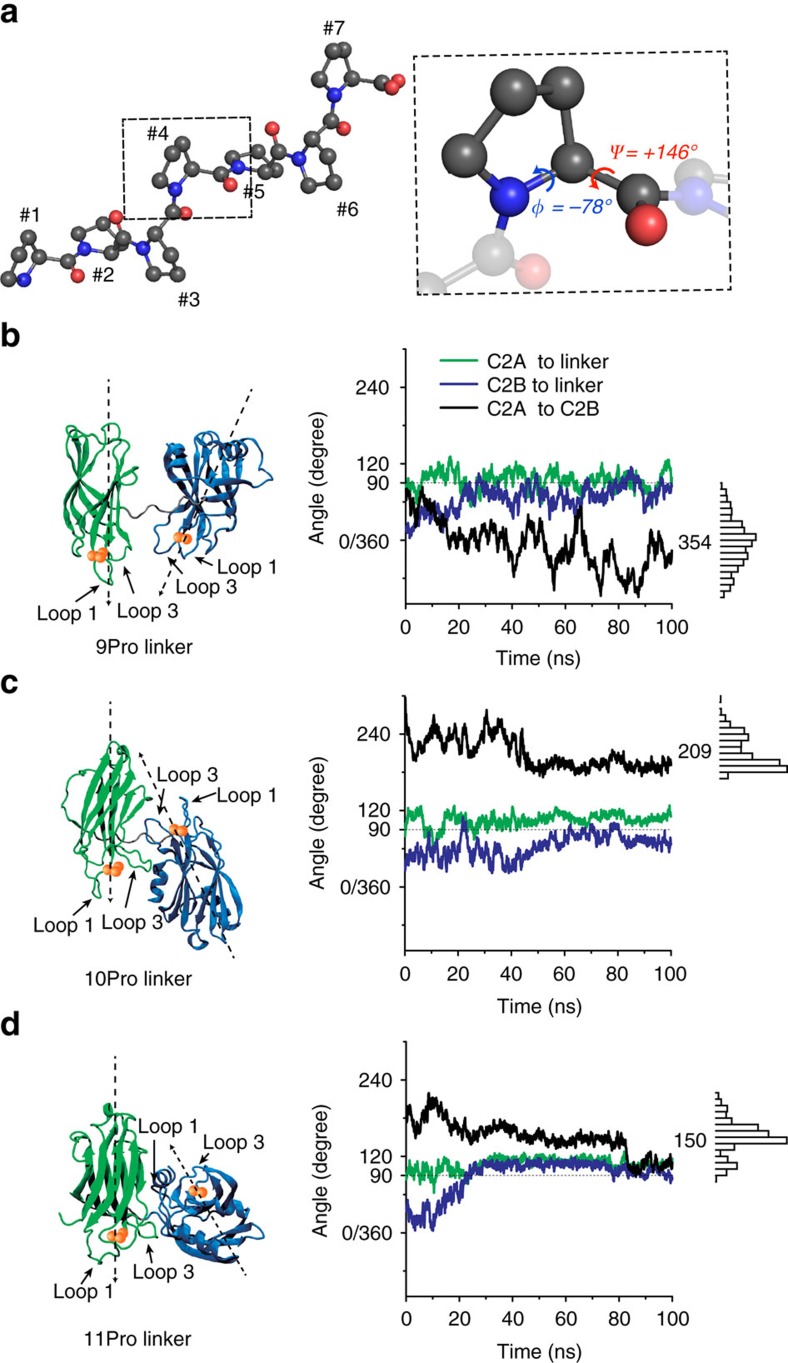
Constraining the relative orientation between the C2A and C2B domains of syt using poly-proline linkers. (**a**) A seven-residue poly-proline (7Pro) helical rod is shown in the left panel; the pitch is three residues per turn of the helix. Right panel: Magnified view in which the dihedral angels, which underlie the periodicity of three, are shown. Nitrogen atoms are represented with blue balls, and the oxygen atoms are represented in Red. (**b**–**d**) The relative orientations of C2A and C2B are predicted to be constrained, and thus altered, by varying the number of residues in the poly-proline linker; these findings are based on the analysis of the **1a-2b** conformer (see definitions and discussion in Supplementary Note 1, Supplementary Figs 7–9 and Supplementary Table 8). Left panels show representative snapshots for the tandem C2-domains connected by poly-proline linkers consisting 9 (**b**), 10 (**c**) or 11 (**d**) residues. Right panels: Results from MD simulations, revealing the predicted relative C2-domain orientations as a function of time. The green and blue traces indicate the relative orientation between the proline linker and either C2A or C2B, respectively. Black traces indicate the relative orientation between C2A and C2B; histograms and numerical averages of relative orientation angles are provided on the right side of each panel.

The major goal of this study was to fix the relative orientation of the C2-domains of syt into specific states, and to determine whether different orientations subserve distinct functions of the protein. To address this, we first replaced the native linker that connects these domains (residues 264–272, SAEKEEQEK) with a nine-residue poly-proline segment (9Pro). As outlined above, poly-proline was used because the side chain of this residue merges into its backbone, so the dihedral angles are relatively stable, with Φ=−78° and Ψ=+146°, resulting in a rigid helix with a periodicity of three^33^,^34^,^35^ (Fig. 1a).

We next sought to determine whether the C2-domains were free to rotate around the proline linker, or whether they were fixed relative to the linker, and hence fixed relative to one-another. We first addressed this question via all-atom MD simulations and metadynamics analysis (detailed in the Methods and Supplementary Notes 1). This analysis revealed that C2A and C2B are highly constrained with respect to the poly-proline linker, and that Ca^2+^-binding loops of the C2A and C2B tend to ‘point' in roughly the same direction in 9Pro mutant form of syt (354°; Fig. 1b). These findings made it possible to systematically alter the relative orientation of these domains by altering the linker one residue at a time, as adding or removing a single residue should have large effects. This prediction was confirmed by simulations using proline linkers of 10 and 11 residues, where the relative angle between C2A and C2B shifted to 209° and 150°, respectively (Fig. 1c,d). Hence, for biochemical and functional studies, we varied the linker from 6 to 9, or in some cases up to 12, prolines (6Pro to 12Pro). Further below, we describe PET experiments that confirm that when the linker is in multiples of three prolines, C2A and C2B point in the same direction.

### Comparison of syt linker mutants in *in vitro* fusion assays

**Figure 2:**
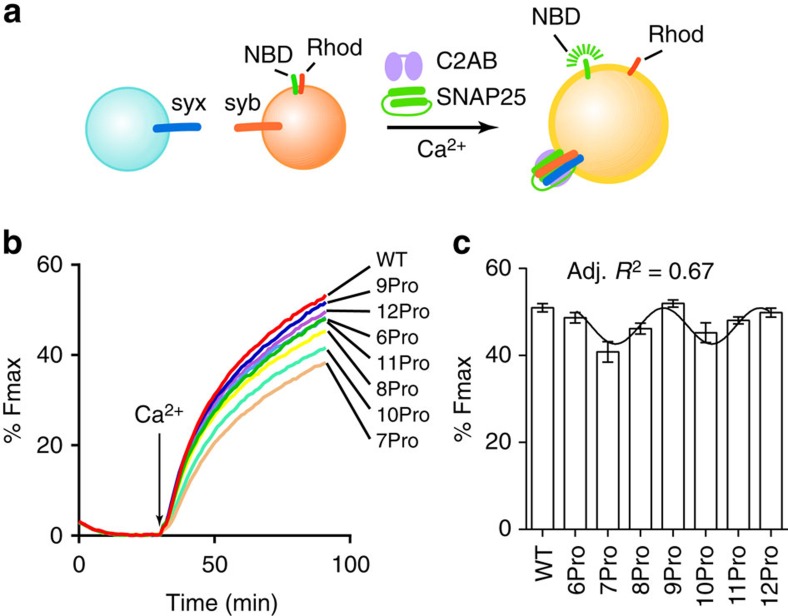
Analysis of syt linker mutant activity in reconstituted fusion reactions. (**a**) Schematic diagram of the reconstituted fusion system; the fluorescence resonance energy transfer donor (NBD) fluorescence increases, because of dequenching, upon fusion. (**b**) Representative traces of reconstituted membrane fusion reactions regulated by WT or linker mutant forms of syt (3 μM); the arrow indicates the addition of Ca^2+^ (1 mM, free). (**c**) The extent of fusion 1 h after addition of Ca^2+^ was plotted and found to be periodic, with a period of three. Data are represented as mean±s.e.m. For each condition, three independent trials were carried out. Data were fitted using a sine wave function with a periodicity of three; an adjusted (Adj.) *R*^2^ value was generated to assess the goodness of the fit.

We examined the impact of the poly-proline linkers on the function of syt using a ‘split t-SNARE' version of the reconstituted, SNARE-mediated, membrane fusion assay^14^,^15^ (Fig. 2a). Briefly, syx was reconstituted, but the other t-SNARE, SNAP-25, was added in a soluble form, such that Ca^2+^·syt must fold SNAP-25 onto membrane-embedded syx to drive fusion. Optimal folding activity requires Ca^2+^·syt·membrane interactions^14^, so this assay system involves both syt-membrane interactions as well as syt-mediated assembly of SNARE complexes. For these, and all biochemical experiments reported here, the cytoplasmic domain of syt was used, and for the electrophysiological studies detailed further below, full-length syt was used.

Upon addition of Ca^2+^, fusion was accelerated by wild-type (WT) syt and each of the linker mutants, but clear differences among the mutants were apparent (Fig. 2b,c). Notably, 6Pro, 9Pro and 12Pro exhibited the greatest activity, followed by 8Pro and 11Pro, whereas 7Pro and 10Pro were the least effective at stimulating fusion. These data were quantified and plotted in Fig. 2c; remarkably, the extent of fusion was well fitted by a wave function with a periodicity of three. This periodicity is consistent with the periodicity of poly-proline rods. Combined with the MD simulations, these results indicate that the ability of syt to drive membrane fusion is strongly influenced by the relative orientation of the tandem C2-domains, and that specific orientations are needed to effectively regulate membrane fusion. This is not a length issue, as the proline rod is fully functional in 12Pro; length becomes an issue when the rod is extended out to 18 prolines, as reported previously^4^. Again, because syt regulates fusion via interactions with both anionic phospholipids^4^,^28^,^38^ and t-SNAREs^14^,^15^,^16^ in the split t-SNARE version of the fusion assay, we examined these interactions in detail in the following section.

### Impact of linker mutations on syt·effector interactions

We assessed the t-SNARE-binding activity of WT and linker mutant forms of syt using a co-flotation assay. Each version of syt was mixed with proteoliposomes harbouring preformed t-SNARE heterodimers in the presence and absence of Ca^2+^; 1,2-dioleoyl-*sn*-glycero-3-[phospho-l-serine] (PS) was omitted to avoid interference by the PS-binding activity of syt^13^. Vesicles were isolated via density gradient centrifugation, subjected to SDS–PAGE, and proteins were visualized by staining with Coomassie blue (Supplementary Fig. 1a). Bound syt was quantified by densitometry (Supplementary Fig. 1b,c). Surprisingly, the t-SNARE-binding activities of all of the linker mutants were as good as, or better than, WT syt. Some degree of periodicity was observed, but this was not well fitted by a wave function with a period of three. Together, these results indicate the loss-of-function observed in the *in vitro* membrane fusion assay was not due to impaired binding of the linker mutants to t-SNAREs. We next examined the interaction of the mutants with anionic phospholipids.

**Figure 3:**
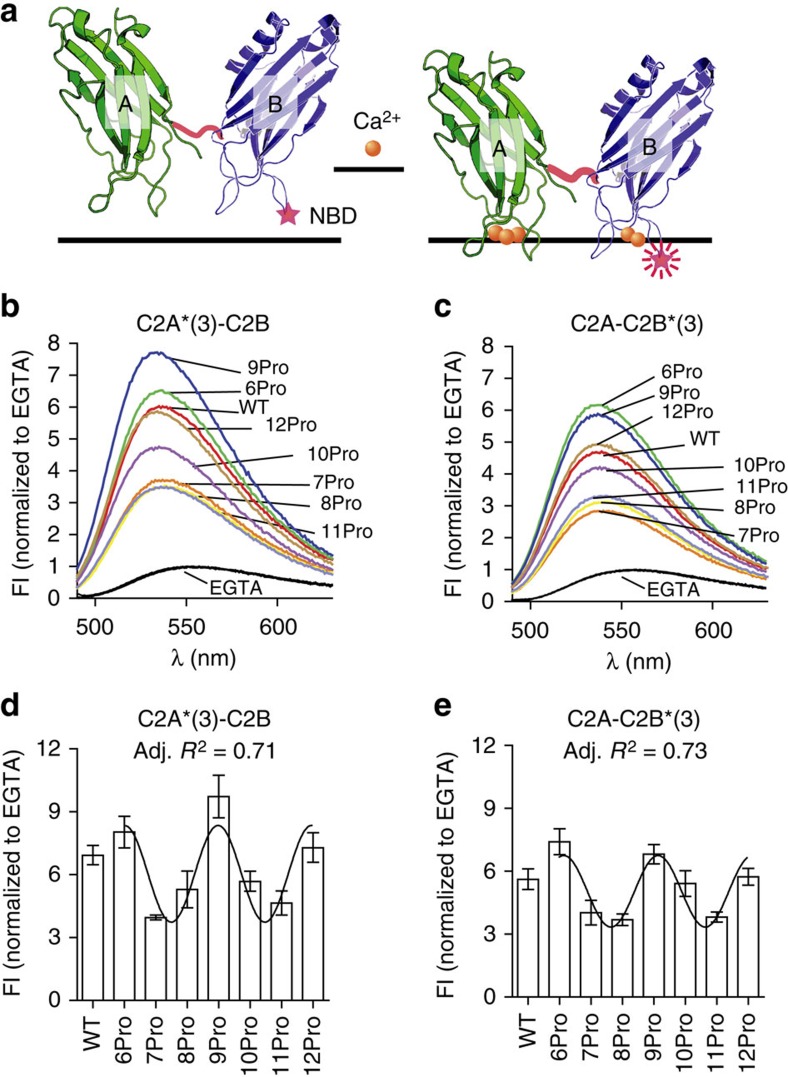
Membrane penetration activity of WT and linker mutant forms of syt. (**a**) Schematic of the *in vitro* membrane penetration assay. The tip of loop 3, in the C2A or C2B domain of syt, was labelled with NBD; the modified proteins are designated as C2A*(3)-C2B and C2A-C2B*(3), respectively^15^,^39^. The NBD probe exhibits an increase in fluorescence intensity (FI), and a blue shift in its emission spectrum, upon Ca^2+^-triggered penetration into lipid bilayers (harbor 15% PS)^15^,^39^. (**b**,**c**) Normalized NBD fluorescence spectra of C2A*(3)-C2B or C2A-C2B*(3) obtained in the absence (0.2 mM EGTA; superimposed black traces) or presence (1 mM) of Ca^2+^ (labelled grey traces). Representative spectra are shown; the protein concentration was 1 μM. (**d**,**e**) Averaged peak values of the NBD fluorescence of C2A*(3)-C2B or C2A-C2B*(3) in 1 mM Ca^2+^, normalized to the signals in EGTA. Data are represented as mean±s.e.m. For each condition, three independent trials were carried out. Data were fitted using a sine wave function with a periodicity of three; an adjusted (Adj.) *R*^2^ value was generated to assess the goodness of the fit.

Upon binding to membranes, the distal tips of the Ca^2+^-binding loops of syt partially insert into the hydrophobic core of the bilayer^4^,^39^. This property has been proposed to drive lipid rearrangements that accelerate fusion as outlined in the Introduction. Indeed, it was recently shown that the membrane penetration activity of syt does in fact constitute a critical step in excitation–secretion coupling^4^. We therefore subjected the linker mutants to *in vitro* membrane penetration assays by labelling the tip of Ca^2+^-binding loop 3 of C2A (F234C) or C2B (I367C) with *N*-(7-nitro-2,1,3-benzoxadiazol-4-yl) (NBD). Labelled protein was incubated with liposomes; upon addition of Ca^2+^, the probe in both C2-domains inserted into bilayers, as evidenced by the observed increase in emission intensity and the shift to shorter wavelengths (Fig. 3b,c)^15^. The fluorescence intensity changes were quantified and found to differ significantly among the linker mutants (Fig. 3d,e). As in the reconstituted fusion reactions, but in contrast to t-SNARE-binding assays, the membrane penetration activity of the linkers yielded a clear periodicity of three, and 6Pro, 9Pro and 12Pro exhibited the greatest activity. These findings were confirmed using a co-sedimentation assay to measure the apparent affinities of each linker mutant for membranes. As with the membrane penetration data, the affinity data also exhibited a periodicity of three (Supplementary Fig. 2). These results further demonstrate that the orientation of the tandem C2-domain of syt strongly influence the biochemical and biophysical properties of the protein. These results confirm the findings from the MD simulations, as a periodicity of three can only occur if the C2-domain constrained.

### Linker mutants maintain the size of readily releasable pool

To determine the physiological consequences that result from altering the relative orientation of the C2-domains of syt in synapses, we used lentivirus to express full-length versions of each mutant in hippocampal neurons derived from syt knockout (KO) mice. All of the linker mutants were correctly targeted to presynaptic boutons, as revealed by their co-localization with synaptophysin (physin), a SV marker (Supplementary Fig. 3).

An important function of syt is to form or maintain the size of readily releasable pool (RRP) of SVs; in syt KO neurons, the RRP, measured via the application of hypertonic sucrose^40^, was significantly reduced^41^. Using the same approach, we observed that each of the poly-proline linker mutants was able to restore the RRP when expressed in syt KO neurons (Supplementary Fig. 4). These observations further show that all of the linker mutants are functional in synapses, and that alterations in the relative orientation of C2A and C2B have no effect on the ability of syt to regulate the RRP.

### Linker mutants differentially regulate two modes of release

**Figure 4:**
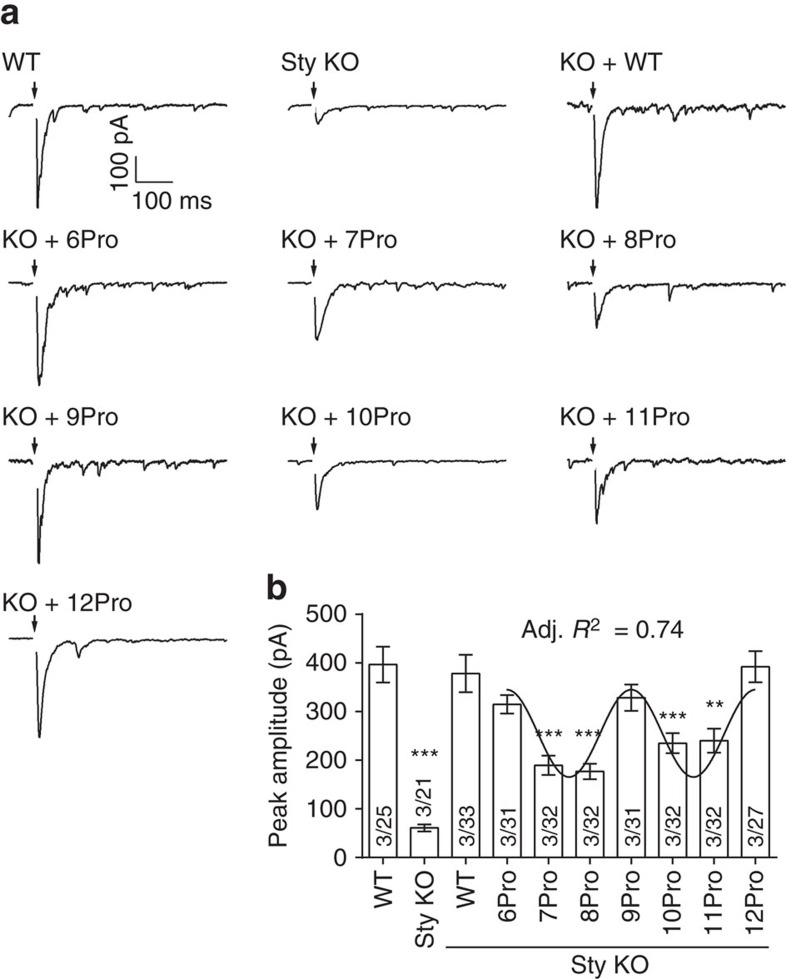
The ability of linker mutants to rescue evoked synaptic transmission exhibits periodicity. (**a**) Representative evoked EPSCs recorded from WT, syt KO and syt KO neurons expressing WT or proline linker mutant forms of syt. Black arrows indicate depolarization. (**b**) Peak amplitudes of evoked EPSCs are plotted. The 6Pro, 9Pro and 12Pro mutants fully rescued the peak amplitude; all other mutants yielded only partial rescue. The degree of rescue was well fitted by a sine wave function with a periodicity of three; an adjusted (Adj.) *R*^2^ value was generated to assess the goodness of the fit. Mean values±s.e.m. are plotted. For each condition, data were collected from 21 to 33 cells from a total of six coverslips, where two coverslips were obtained from each of three independent litters of mice. Recording were made from 3 to 7 cells per coverslip. **P<0.001, ***P<0.001 versus WT, one-way analysis of variance (ANOVA) followed by Tukey's multiple comparisons test. The number of independent litters, *N*, and the number of cells, *n*, are indicated in the bar graph as *N*/*n*. Full results of the one-way ANOVA are in Supplementary Table 4.

In the next series of experiments, we measured the ability of the linker mutants to rescue the loss of rapid evoked synaptic transmission in syt KO neurons. As expected, fast release was largely absent in syt KO neurons, but was fully rescued by WT syt (Fig. 4). All of the linker mutant forms of syt were able to restore the time latency between presynaptic stimulation and peak of the EPSCs (time-to-peak; Supplementary Table 1). However, the degree of rescue of the EPSC peak amplitude differed significantly among the mutants (Fig. 4b). Because the size of the RRP was identical among all conditions (Supplementary Fig. 4), differences in EPSC amplitude were due to differences in the ability of the linker mutants to trigger SV exocytosis and were not secondary to reductions in releasable vesicles. Importantly, the extent of fast neurotransmitter release in synapses exhibited a periodicity of three, identical to the periodicity observed for *in vitro* fusion assays and syt–membrane interactions. Briefly, 6Pro, 9Pro and 12Pro completely rescued the fast phase of neurotransmission, whereas the other constructs yielded partial recovery of evoked release (Fig. 4b). Together with the findings above, we conclude that the relative orientation between C2A and C2B is crucial for syt to function as a fast Ca^2+^ sensor during synaptic transmission, and suggest that syt drives fusion when its C2-domains point in the same direction.

**Figure 5:**
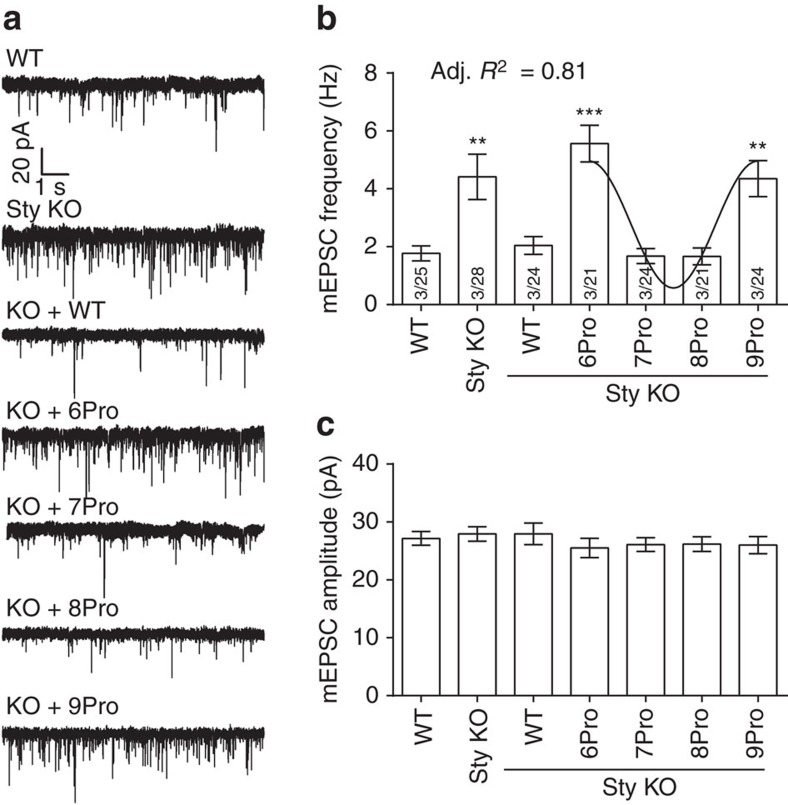
Linker mutants yield differences in spontaneous release rates. (**a**) Representative mEPSC traces recorded from WT, syt KO and syt KO neurons expressing WT or proline linker mutant forms of syt. The frequency (**b**) and amplitude (**c**) of mEPSCs are plotted as mean value±s.e.m. The 6Pro and 9Pro mutants were unable to clamp spontaneous fusion, whereas 7Pro and 8Pro had robust clamping activity. For each condition, data were collected from 21 to 28 cells from a total of six coverslips, where two coverslips were obtained from each of three independent litters of mice. Recording were made from 3 to 7 cells per coverslip. **P<0.001, ***P<0.001 versus WT, one-way analysis of variance (ANOVA) followed by Tukey's multiple comparisons test. The number of independent litters, *N*, and the number of cells, *n*, are indicated in the bar graph as *N*/*n*. One-way ANOVA results are provided in Supplementary Tables 5 and 6.

We next examined the abilities of the linker mutants to clamp spontaneous release (that is, miniature excitatory postsynaptic currents; mEPSC). mEPSCs were recorded from syt KO neurons expressing 6Pro, 7Pro, 8Pro and 9Pro mutants of syt, which cover a complete period of the relative angle between C2-domains. Strikingly, a periodicity of three was also found for the frequency of mEPSC events. As shown in Fig. 5a,b, syt KO neurons expressing 6Pro and 9Pro exhibited no differences in mEPSC frequency as compared with control syt KO neurons, whereas 7Pro and 8Pro clamped spontaneous synaptic transmission to a same extent as WT syt. The amplitude of the mEPSCs were the same for WT and all of the mutants (Fig. 5c).

### Structural states of syt that subserve distinct functions

**Figure 6:**
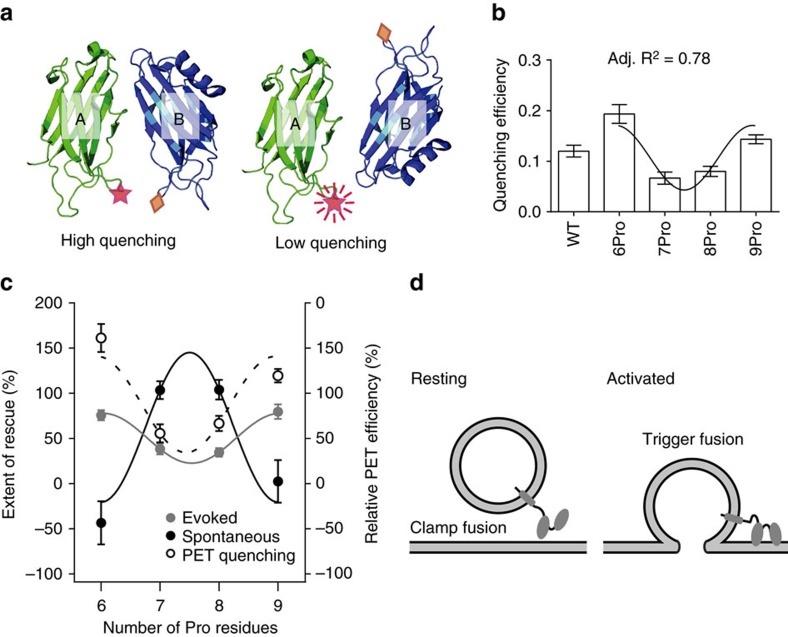
Distinct relative C2-domain orientations underlie syt regulation of evoked and spontaneous release. (**a**) Illustration showing the PET quenching assay, showing the quenching Trp (diamond) and the BODIPY fluorophore (star) that is quenched, in the membrane penetration loops of C2A and C2B, respectively. (**b**) The quenching efficiencies were calculated and plotted for the WT and 6–9Pro mutants; 0.5 μM protein was used in each condition. Data were fitted using a sine wave function with a periodicity of three; an adjusted (Adj.) *R*^2^ value was generated to assess the goodness of the fit. The most efficient quenching was observed for 6Pro and 9Pro, so the C2-domains in these two constructs point in the same direction; for 7Pro and 8Pro, the C2-domains are not in a parallel relative orientation. Data are represented as mean±s.e.m.; for each condition, three independent trials were carried out. (**c**) Reciprocal abilities of the syt linker mutants to clamp spontaneous release and to drive evoked synaptic transmission. The amplitude of evoked EPSCs and the frequency of mEPSC were used to evaluate the function each linker mutant. Data were normalized using values obtained from WT (100%) and syt KO neurons (0%). Again, the results were fitted with a sine wave function with a periodicity of three; an adjusted *R*^2^ value was generated to assess the goodness of the fit. For completeness, the PET quenching data were normalized and overlaid onto this plot, to reveal the relative orientations that underlie the regulation of evoked versus spontaneous release. (**d**) Under resting conditions, C2A and C2B point to different directions to clamp fusion; when activated by Ca^2+^, C2A and C2B switch to a parallel configuration to trigger SV exocytosis.

The evoked and spontaneous release data for the 6-9Pro linker mutants were normalized relative to WT, and plotted in Fig. 6c, revealing a reciprocal relationship between the mutants that drove evoked release and the mutants that inhibited spontaneous release. Linker mutants that drive efficient evoked release are less effective at clamping spontaneous release, whereas mutants that effectively clamp spontaneous release fail to drive efficient evoked transmission. These results demonstrate that syt can switch between at least two different functional states via changes in the relative orientation of its tandem C2-domains.

In the final series of experiments, we used PET^42^ to experimentally address the relative orientations of the tandem C2-domains in WT and the 6–9Pro linker mutants. We labelled the same positions used for the membrane penetration assays above (that is, membrane penetration loop 3 in both C2A and C2B). However, in this case, the engineered Cys residue in C2A was modified with the boron-dipyrromethene dye BODIPY FL (Bpy); the PET quencher, Trp, was placed in C2B (Fig. 6a). The PET quenching efficiency for the 6-9Pro linker mutant constructs were determined and the results revealed a periodicity of three (Fig. 6b). Maximal quenching was observed for 6 and 9Pro, whereas the 7 and 8Pro yielded little quenching. As Trp-mediated PET quenching occurs on the 0.5–1.5 nm distance scale, these results indicate that in the 6 and 9Pro mutants, the tandem C2-domains are in the closest proximity, and hence point in the same direction, whereas 7 and 8Pro are further apart so the C2-domains point in different directions.

We then normalized the PET quenching data, and overlaid these results onto the evoked and spontaneous release plots shown in Fig. 6c. From this comparison, it is apparent that syt drives evoked release when its tandem C2-domains point in the same direction (for example, 6Pro, 9Pro, and by inference, 12Pro), and syt clamps fusion when the C2-domains point in different directions.

## Discussion

Because the individual C2-domains of syt do not appear to undergo large conformational changes^8^,^9^, we propose that changes in the relative disposition of the C2-domains underlie the ability of syt to switch between functional states. In this model, large conformation changes in syt can be achieved without significant structural changes within the individual domains. Previous X-ray crystallography studies shed some light on this question. In one structure of syt, the Ca^2+^-binding loops of C2A and C2B were roughly perpendicular to one-another^31^, suggesting that one C2-domain might penetrate the plasma membrane while the other C2-domain could penetrate a fusion neck or stalk^43^. However, in another crystal structure, in this case of the cytoplasmic domain of syt3, the tandem C2-domains formed a shallow v-shape, with the Ca^2+^-binding loops of C2A and C2B largely pointing towards one-another^29^. In contrast, two other groups proposed a model in which C2A and C2B have an ‘antiparallel conformation' such that the Ca^2+^-binding loops of C2A and C2B point in opposite directions^27^,^44^. In another study, based on nuclear magnetic resonance measurements, it was concluded that the relative orientation is dynamic, but that there might be a limited set of preferred states^30^. In a newer nuclear magnetic resonance study, it was demonstrated that the ‘parallel orientation' of the C2-domains of syt is the most favourable conformation when engaging membranes (nanodiscs) in the presence of Ca^2+^ (ref. 45). Finally, kinetics studies showed that, in response to Ca^2+^, C2A and C2B simultaneously penetrate the target membrane, suggesting that the tandem C2-domains point in the same direction^24^,^39^. Given the distinct views concerning the relative disposition of the tandem C2-domains of syt, we addressed the debate by replacing the nine-residue linker connecting the tandem C2-domains of syt with a series of poly-proline linkers, which constrain the relative orientation of C2A and C2B to specific, stable, states. We then carried out biochemical and cell-based experiments to discern the functionality of each of these constrained forms of the protein.

Strikingly, adding or removing single residues from the nine-residue poly-proline rod revealed a clear periodicity of three in all but one of the assays that were carried out; the lone exception was the t-SNARE-binding activity of the linker mutants. The periodicity was evident in *in vitro* reconstituted fusion assays, membrane penetration/binding assays, PET quenching experiments and electrophysiological measurements of evoked and spontaneous release. For example, among all the linker mutants, the 6Pro, 9Pro, 12Pro fully rescued evoked synaptic transmission, whereas the other linker mutants yielded impaired EPSCs. The MD simulations, in conjunction with our experimental data, indicate that in 6Pro, 9Pro and 12Pro, the tandem C2-domains of syt point the same direction; syt drives efficient SV exocytosis when its tandem C2-domains are in a parallel configuration.

We compared evoked with spontaneous release regulated by each of the linker mutants, and a clear reciprocal relationship emerged (Fig. 6c). These results help to explain how syt is able to regulate distinct forms of exocytosis: at rest, the C2-domains point in different directions to inhibit spontaneous SV exocytosis. Then, in response to Ca^2+^, syt changes states via large-scale alterations in the disposition of its tandem C2-domains; these relative movements allow both domains point in the same direction to interact with the plasma membrane to drive fusion (Fig. 6d)^24^,^39^,^46^.

There are a variety of proteins that contain tandem C2-domains, and myriad other structural motifs. The rigidity of poly-proline rods can be further exploited to explore the functional interactions between these domains, and to alter their relative orientations, as described here.

## Methods

### Molecular biology

cDNA encoding rat syt^47^ was provided by T.C. Südhof (Stanford University); the D374 mutation was corrected by replacement with a glycine residue^48^. The indicated poly-proline linker mutant forms of syt were generated via PCR using the overlapping primer method^49^. Full-length versions were subcloned into a modified pLOX vector (Addgene)^50^; fragments encoding the cytoplasmic domains were subcloned into pGEX-4T vectors. For site-directed labelling, the lone native cysteine (Cys277) in the cytoplasmic domain of syt was substituted with an alanine. Then, a single cysteine was introduced at Phe234 of C2A, or Ile367 of C2B. Point mutations were generated using a QuikChange Site-Directed Mutagenesis Kit (Stratagene). WT and linker mutant forms of the cytoplasmic domain of syt (residues 96–421) were expressed as glutathione *S*-transferase fusion proteins, purified using glutathione–Sepharose beads (GE healthcare), and cleaved with thrombin to remove glutathione *S*-transferase^23^. cDNA encoding full-length rat syntaxin-1A, mouse SNAP-25B and mouse synaptobrevin-2 was provided by R.H. Scheller (Genentech), M.C. Wilson (Univ. New Mexico) and J.E. Rothman (Yale University), respectively. Each SNARE cDNA was subcloned into a pTrcHis vector (Invitrogen), resulting in an N-terminal His_6_-tag that was used for purification^14^. The original sequences of all primers used in this study are provided in Supplementary Table 2.

### Hippocampal neuronal culture and viral infection

Syt KO mice were obtained from Jackson Laboratory, and a colony was established. Primary cultures of hippocampal neurons were prepared from syt KO mice, or their WT littermates, at postnatal day 0, in accordance with the guidelines of the National Institutes of Health, as approved by the Animal Care and Use Committee at the University of Wisconsin. Briefly, hippocampi were isolated from WT and KO mice, washed with Hank's buffered salt solution and digested for 30 min in the same buffer plus 0.25% (w/v) trypsin and 2.21 mM EDTA at 37 °C. Hippocampi were transferred to Dulbecco's Modified Eagle Medium supplemented with 10% fetal bovine serum (Hyclone), and then dissociated mechanically. The dissociated neurons were plated on 12 mm glass coverslips (Warner Instruments), coated with poly-D-lysine, at a density of 25,000–40,000 cells per cm^2^. Neuronal cultures were maintained in Neurobasal-A medium, supplemented with B27 and GlutaMAX (Invitrogen), at 37 °C in a 5% CO_2_ humidified incubator.

To generate viruses, the syt pLOX constructs were co-transfected into HEK-293T cells (American Type Culture Collection, not authenticated, not tested for micoplasma) with two lentiviral packaging vectors (vesicular stomatitis virus G glycoprotein and Delta 8.9)^50^. Three days after transfection, lentivirus was harvested from the media by centrifugation at 126,000*g* for 2 h. Neuronal cultures were infected with virus at 5 DIV (days *in vitro*). The infection rate was ∼90%, as estimated by the co-expression of GFP.

### Immunocytochemistry

At 13–15 DIV, neuronal cultures were fixed with 4% paraformaldehyde (w/v) in PBS for 15 min, permeabilized in 0.1% Triton X-100 (v/v) for 10 min and then blocked in 10% bovine serum albumin (w/v) plus 0.1% Triton X-100 (v/v) for 30 min. Coverslips were incubated with a mouse monoclonal antibody against syt (mAb48, diluted 1:1,000, Developmental Studies Hybridoma Bank)^51^ and, to visualize nerve terminals, a polyclonal guinea pig antibody against physin (Cat. No. 101004, diluted 1:1,000; Synaptic System) for 2 h. Coverslips were washed three times with PBS and incubated with Alexa 488-tagged anti-mouse and Alexa 594-tagged anti-guinea pig secondary antibodies (Cat. No. 115–547–187 and 706–586–148; diluted 1:400; Jackson ImmunoResearch Laboratories) for 1 h. Coverslips were again washed three times and mounted in Fluoromount (Southern Biotechnology Associates). Images were acquired using an Olympus FV1000 confocal microscope with a × 60 1.40NA oil objective, using fixed laser intensity and gain settings. To quantify the co-localization of each of the syt constructs with physin, the Mander's coefficients (syt to physin) for each image pair were calculated using the JACoP plug-in (ref. 52) in ImageJ software.

### Electrophysiology

Whole-cell patch clamp recordings were obtained from cultured hippocampal neurons at 13–17 DIV. During recordings, neurons were continuously perfused with bath solution (128 mM NaCl, 30 mM glucose, 5 mM KCl, 2 mM CaCl_2_, 1 mM MgCl_2_, 50 μM D-AP5, 20 μM bicuculline and 25 mM HEPES, pH 7.3). The recording pipettes were pulled to a resistance of 3–5 MΩ from glass capillaries (Warner Instruments) and filled with standard intracellular solution (130 mM K-gluconate, 1 mM EGTA, 5 mM Na-phosphocreatine, 2 mM Mg-ATP, 0.3 mM Na-GTP, 5 mM QX-314 and 10 mM HEPES, pH 7.3). Neurons were voltage clamped at −70 mV via the recording pipettes using a MultiClamp 700B amplifier (Molecular Devices). Only whole-cell patches with series resistances <15 MΩ were used for recording.

For evoked EPSCs, the presynaptic neuron was depolarized by a 1-ms voltage step (40 V) using a bipolar electrode pulled from theta glass capillary (Warner Instruments) and filled with bath solution. For the RRP measurement, the field of view (at × 40) around the patch-clamped neuron was locally perfused with bath solution plus 500 mM sucrose for 10 s using a Picospritzer III (Parker), releasing the entire RRP of all connected presynaptic boutons. For mEPSCs, 1 μM tetrodotoxin was present in the bath solution. All experiments were performed at room temperature (RT). Data were acquired at a sampling rate of 10 kHz and filtered at 2.8 kHz using the pClamp (Molecular Devices) software. Off-line analysis was performed using the Clampfit (Molecular Devices) and Igor (WaveMetrics) software. The D-AP5, bicuculline, tetrodotoxin and QX-314 were purchased from TOCRIS Bioscience. All recordings were obtained from randomly selected cells.

### Liposomes

Synthetic 1, 2-dioleoyl-*sn*-glycero-3-phospho-l-serine (phosphatidylserine, PS), 1-palmitoyl-2-oleoyl-*sn*-glycero-3-phosphoethanolamine (phosphatidylethanolamine, PE), 1-palmitoyl-2-oleoyl-*sn*-glycero-3-phosphocholine (phosphatidylcholine, PC), 1,2-dipalmitoyl-*sn*-glycero-3-phospho-ethanolamine-*N*-(7-nitro-2–1, 3-benzoxadiazol-4-yl) (NBD-PE) and N-(lissamine rhodamine B sulfonyl)-1, 2-dipalmitoyl-*sn*-glycero-3-phosphoethanolamine (rhodamine-PE) were obtained from Avanti Polar Lipids. Protein-free SUVs (small unilamellar vesicles) were prepared as follows: lipids (15% PS+30% PE+ 55% PC or 25% PS+30% PE+45% PC, as indicated) were mixed and dried under nitrogen, and lyophilized for 1 h. The dried lipid film was resuspended in Tris buffer (50 mM Tris, pH 7.4, 150 mM KCl) and extruded 20 times through 100 nm polycarbonate filters. SNARE-bearing SUVs were prepared^14^ using 15% PS+30% PE+55% PC for t-SNARE vesicles (Tr) and 15% PS+27% PE+55% PC+1.5% NBD-PE+1.5% rhodamine-PE for v-SNARE vesicles (Vr).

### Labeling of the cytoplasmic domain of syt with NBD

For labelling syt with NBD for membrane penetration experiments, the appropriate purified cysteine mutants were adjusted to ∼10 μM in Tris buffer (50 mM Tris, pH 7.4, 150 mM KCl). NBD (Molecular Probes) was then added drop-wise to each protein solution to reach a final concentration of ∼150 μM; samples were incubated at RT for 2 h with rotation, and free dye was removed by extensive dialysis against Tris buffer (50 mM Tris, pH 7.4, 150 mM KCl). The NBD concentration was determined by absorbance using a molar extinction coefficient of 25,000 M^−1^ cm^−1^ at 480 nm. The protein concentration was determined by SDS–PAGE and staining with Coomassie blue, using BSA as a standard. The labelling stoichiometry was 0.7–0.9 mol of dye per mole of protein.

### Fluorescence measurements

Membrane penetration assays were performed at RT using a PTI QM-1 fluorometer and FELIX software (Photon Technology International). For membrane penetration assays, NBD-labelled protein was mixed with SUVs (113 μM total lipid) in Tris buffer (50 mM Tris, pH 7.4, 150 mM KCl), in the presence of either EGTA (0.2 mM final concentration) or Ca^2+^ (1 mM final free concentrations). NBD was excited at 470 nm, and the emission spectra (490 to 630 nm; 4-nm slits) were corrected for blank and instrument response.

### Co-sedimentation assays

WT or linker mutant cytoplasmic domains of syt (4 μM) were mixed with PS-bearing liposomes (at the indicated concentrations) in Tris buffer (50 mM Tris, pH 7.4, 150 mM KCl), in the presence of 1 mM Ca^2+^ or 0.2 mM EGTA, and then centrifuged at 170,000*g* at 4 °C for 45 min in a Beckman Optima MAX-E (Beckman Coulter) tabletop ultracentrifuge. The supernatant from each sample was collected and analysed by SDS–PAGE and Coomassie blue staining. Bands were quantified by densitometry to reveal the amount of free protein, and these data were used to calculate the amount of bound protein, which was then plotted versus the PS concentration (mM); apparent *K*_d_ values were determined by fitting the data with ‘one-site-specific binding curves' using Prism 6 (Graphpad Software). An uncropped image of entire gel is provided in Supplementary Fig. 5.

### Co-flotation assays

One hundred-microlitre reactions contained 11 μM WT or linker mutant forms of the cytoplasmic domain of syt, 45 μl of PS-free t-SNARE vesicles and reconstitution buffer (25 mM HEPES-KOH, pH 7.4, 100 mM KCl, 10% glycerol, 1 mM dithiothreitol)^13^. Samples contained either 0.2 mM EGTA or 1 mM free Ca^2+^, and were incubated at RT for 45 min with shaking, loaded onto an Accudenz gradient, centrifuged at 287,000*g*, and the vesicles were collected at the 0–30% Accudenz interface. Samples were then subjected to SDS–PAGE and stained with Coomassie blue; protein bands were quantified by densitometry. Uncropped images of entire gels are provided in Supplementary Fig. 5.

### *In vitro* fusion assays

‘Split t-SNARE' fusion assays were carried out^15^; in this variant of the fusion assay, syx is reconstituted but SNAP-25 is added in a soluble form. This configuration of the assay bypasses the ability of syt to stimulate fusion by merely aggregating v- and t-SNARE vesicles when the t-SNAREs, syx and SNAP-25, are reconstituted as pre-formed heterodimers. Rather, Ca^2+^·syt must direct the folding of SNAP-25 onto syx, resulting in the assembly of functional *trans* SNARE complexes^14^, thus driving fusion. Samples were incubated at 37 °C for 30 min in the presence of 0.2 mM EGTA, followed by addition of Ca^2+^ (1 mM final free concentration). Fusion was monitored, as dequenching of NBD fluorescence, for an additional 1 h. The detergent, β-D-maltoside (0.5% w/v), was then added, resulting in ‘infinite' dilution of the NBD-rhodamine fluorescence resonance energy transfer pair, yielding the maximum fluorescence signal used to normalize the data.

### PET quenching assay

A single Cys was placed at position F234C in the cytoplasmic domain of syt as described above. A second version was constructed, in which the F234C mutant also included a second mutation, I367W. The Cys residue, in both constructs, was labelled using a fivefold molar excess of the maleimide derivative of BODIPY FL (Life Technologies), for 2 h at 25 °C in labelling buffer (20 mM HEPES, pH 7.4, 100 mM KCl, 10% glycerol). The reaction was quenched by addition of 5 mM dithiothreitol, and free dye was removed by gel filtration on a Superdex 200 10/300 column (GE Healthcare) in labelling buffer. The labelling efficiency was ∼80% for both constructs. PET measurements, using the Trp in C2B to quench the fluorescence of the BODIPY in C2A, were carried out^42^. Briefly, fluorescence measurements were performed in reconstitution buffer at 25 °C; samples were excited at 470 nm, and the emission was collected from 500 to 600 nm. The efficiency of quenching was calculated by comparing the BODIPY fluorescence intensity in the absence and presence of the Trp quencher.

### Simulations and free energy computations with metadynamics

All MD simulations were performed using NAMD 2.9 (ref. 53) and the CHARMM22 force field^54^,^55^ for proteins. Simulations were carried out in the constant pressure-temperature ensemble at 1 atm and 298 K using Nosé-Hoover and Langevin piston pressure coupling protocols. Particle Mesh Ewald was used for the electrostatics and Lennard–Jones interactions were treated with a cutoff of 12 Å and a switching function turned on at 10 Å.

To characterize the rotational flexibility of the poly-proline linker with respect to the C2-domains, we performed two-dimensional metadynamics simulations for individual C2-domains connected to a nine-proline linker (that is, C2A-9Pro and C2B-9Pro)^56^ to compute the free energy map as a function of the two dihedral angles defined in Supplementary Fig. 6. These angles were chosen based on previous studies that characterize conformational isomerization of proline^57^, and different dihedral angles were used for C2A-9Pro and C2B-9Pro because the 9Pro peptide is connected to C2A at its N-terminus, whereas the linker is connected to C2B at its C-terminus. The built-in version of metadynamics in NAMD 2.9 was used. The Gaussian width was set to 9.0° and the Gaussian height was 0.07 kcal mol^−1^; Gaussian was added every 0.2 ps. Convergence of the metadynamics simulation was monitored based on the distribution of the collective variables. The reported free energy maps in Supplementary Fig. 7 were obtained with 70 ns of metadynamics for each C2-domain.

For the starting structure, the coordinates for isolated C2A or C2B were extracted from PDB entry 2R83 (ref. 31); Ca^2+^ ions were not included in the model. The native linker, SAEKEEQEK, was replaced with a poly-proline peptide using CHARMM c37a1 (ref. 58). The system was then solvated with TIP3P water and 0.15 M of NaCl; the average box size during the simulation was 80 × 80 × 80 Å^3^.

Following the metadynamics simulations for the individual domains, stable conformers were identified based on the computed free energy maps, and these were used to build models for C2A-C2B connected with a poly-proline linker (C2A-*n*Pro-C2B, where *n* is the number of proline residues); three linker lengths (*n*=9, 10 and 11) were simulated. C2A-*n*Pro-C2B were solvated with TIP3P water and 0.15 M of NaCl; the average box size during the simulation was 110 × 110 × 110 Å^3^. The systems were simulated for at least 70 ns. The relative orientations of the Ca^2+^-binding loops relative to poly-proline linker in the C2-domains were monitored and shown in Supplementary Fig. 8.

### Statistical analysis

The sine wave traces, superimposed on the bar graph in Figs 2, 3, 4, 5, 6, were generated by fitting the data using sine wave functions, with a periodicity of three, using Origin 9 (OriginLab), as follows:


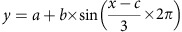


in which *y* corresponds to the measured variable, *x* is the number of proline residues in the poly-proline linker and *a*, *b* and *c* are the offset, amplitude and phase parameters, respectively. The detailed results are listed in Supplementary Table 3.

For the *in vitro* biochemical studies, three independent trials were carried out. No further statistical analysis, besides fitting with the sine wave function, was carried out.

In all the electrophysiological experiments, the number of recorded cells, coverslips and independent litters of mice are detailed in the Figure Legends. One-way analysis of variance followed by Tukey's multiple comparisons test were carried out to compare differences among groups. Detailed results of all statistical analysis are provided in the Supplementary Tables 4–7. We did not perform any statistical tests to justify the sample sizes; however, the sample sizes in this study follow the standard used in this field^4^,^41^,^59^. We did not asses the normality of the results; however, we present the electro-physiological data as scatter plots so the variance of the raw data is evident.

Experiments were not carried out blind.

## Additional information

**How to cite this article:** Bai, H. *et al*. Different states of synaptotagmin regulate evoked versus spontaneous release. *Nat. Commun.* 7:10971 doi: 10.1038/ncomms10971 (2016).

## Supplementary Material

Supplementary Information Supplementary Figures 1-9, Supplementary Tables 1-8, Supplementary Note 1 and Supplementary References
