# Supplementary material for: Different states of synaptotagmin regulate evoked versus spontaneous release
Source: Nat Commun. 2016 Mar 22;7:10971. doi: 10.1038/ncomms10971 (PMC4804166; doi:10.1038/ncomms10971)
Supplement: Supplementary Information — Supplementary Figures 1-9, Supplementary Tables 1-8, Supplementary Note 1 and Supplementary References [file ncomms10971-s1.pdf]

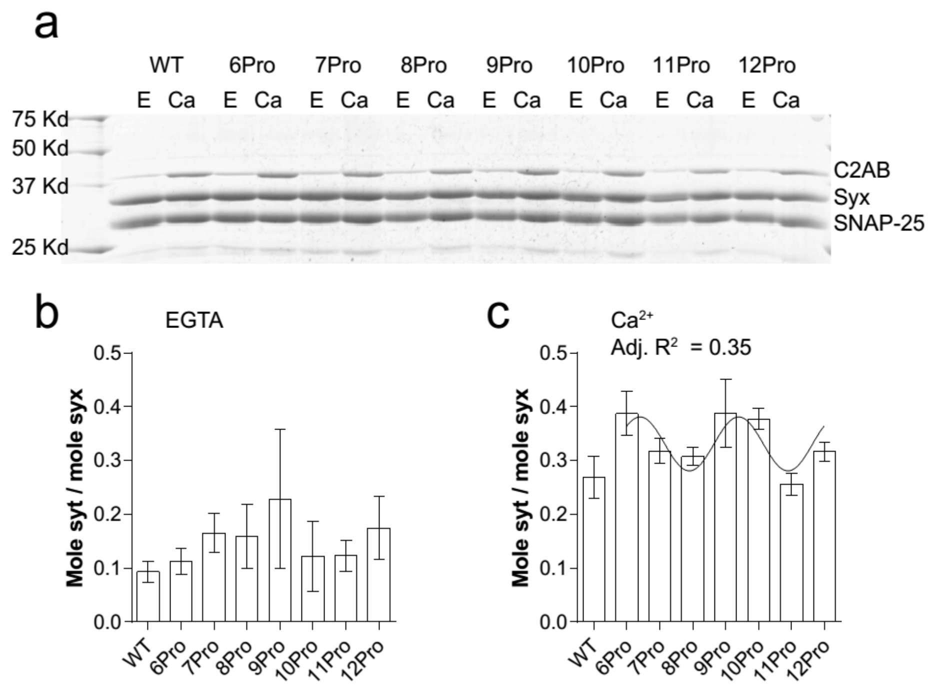

Supplementary Figure 1. t-SNARE binding activity of syt poly-proline linker mutants. (**a**) t-SNARE binding activity, defined as the mole of WT or mutant syt bound per mole of syx, was measured using a co-flotation assay. The final $[Ca^{2+}]_{free}$ was 1 mM; samples lacking Ca^{2+} contained 0.2 mM [EGTA]. Three independent experiments were carried out; a representative gel is shown. An uncropped image of and entire gel is provided in Supplementary Fig. 5a. Data from panel a were quantified by densitometry, and the extent of binding in both EGTA (**b**) and Ca^{2+} (**c**) was plotted. The plot for Ca^{2+} dependent t-SNARE binding was overlaid with a sine wave (**c**); while some periodicity was observed, it was not well fitted using a period of three. Data are represented as mean ± SEM. Data were fitted using a sine wave function with a periodicity of three as described in Methods; an adjusted R^2 value was generated to assess the goodness of the fit.

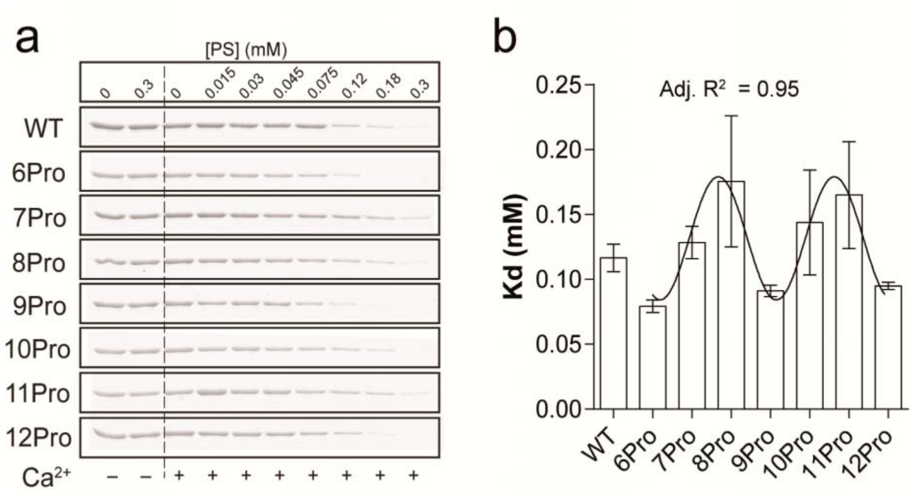

Supplementary Figure 2. Changes in the poly-proline linker affect the Ca^{2+} promoted PS binding activity of syt. (**a**) Binding of WT or linker mutant forms of syt cytoplasmic domain to PS-bearing liposomes was monitored using a co-sedimentation assay; depletion of the supernatant was monitored via SDS-PAGE and staining with Coomassie blue, and these data were used to calculate the amount of bound material, in the absence (-; 0.2 mM EGTA) or presence (+) of 1 mM $[Ca^{2+}]_{free.}$ An uncropped image of entire gels are provided in Supplementary Fig. 5b. No molecular makers were used because only one purified protein was loaded onto each gel. (**b**) Apparent K_d values for syt•membrane interactions, were determined as described in Methods. Data were fitted using a sine wave function with a periodicity of three described in Methods; an adjusted R^2 value was generated to assess the goodness of the fit. Data are represented as mean ± SEM. For each condition, three independent trials were carried out.

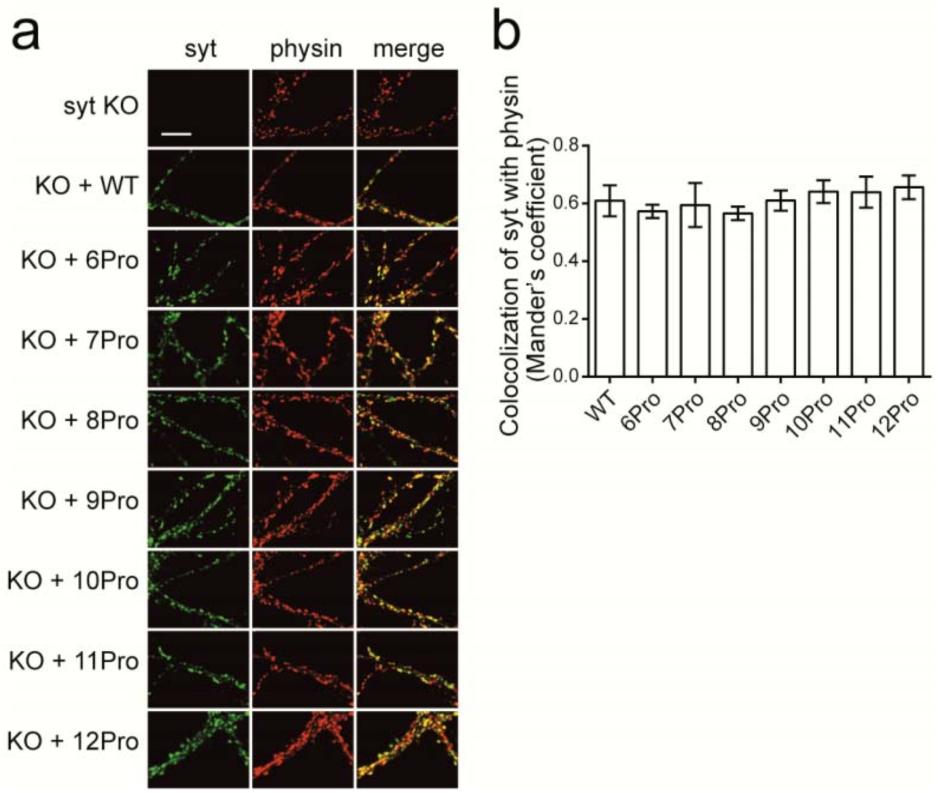

Supplementary Figure 3. Proline linker mutant forms of syt are properly targeted to synapses. (**a**) Full-length WT or proline linker mutant forms of syt were expressed in syt KO hippocampal neurons via lentiviral infection. Neurons were immunostained at 13 ~ 15 DIV using anti-syt (green) and anti-physin (red) antibodies. Shown are representative confocal images. Scale bar = 10 μm. (**b**) The degree of colocalization was quantified and plotted; all syt constructs were efficiently targeted to synapses. Data are represented as mean ± SEM. For each condition, n = 6, two independent cultures from independent litters of mice were examined, and three independent regions from each coverslip were analyzed. No significance was detected using one-way ANOVA, F = 0.5024, p = 0.8271.

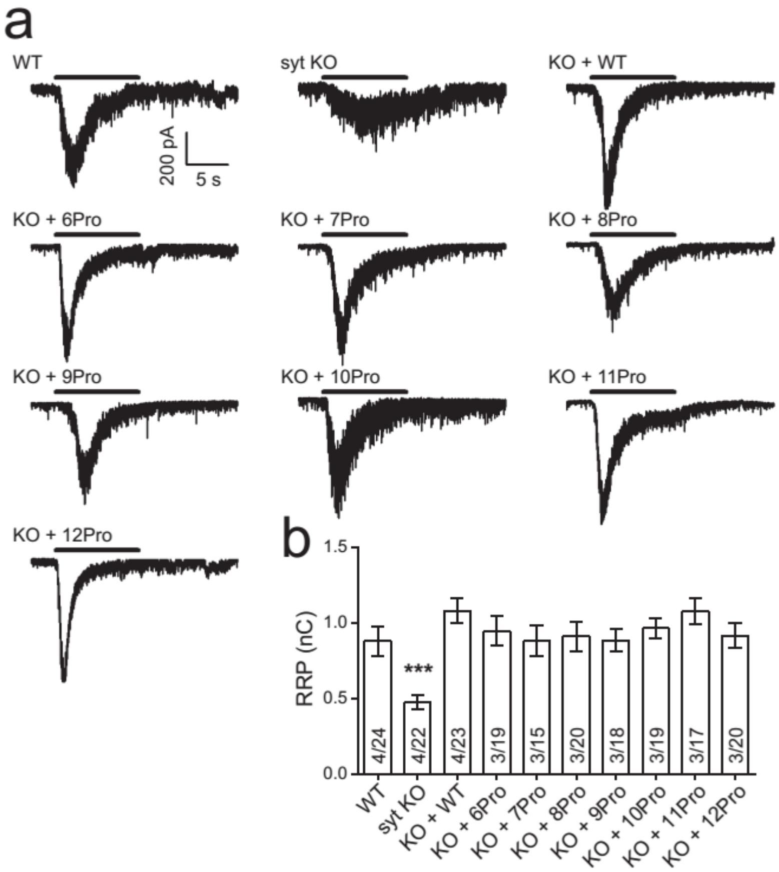

Supplementary Figure 4. All of the proline linker mutant forms of syt fully rescued the size of the RRP in syt KO neurons. (**a**) Representative EPSCs, elicited by perfusion of hypertonic sucrose (500 mM), were recorded from WT, syt KO, and syt KO neurons expressing full-length WT or proline linker mutant forms of syt; the black bars indicate the application of sucrose. (**b**) RRP size was quantified by integrating the sucrose-driven EPSCs. All of the proline linker mutants rescued the size of the RRP to a similar extent as WT syt. Data are represented as mean ± SEM. *** $p < 0.001$ versus WT, one-way ANOVA followed by Tukey's multiple comparisons test. For each condition, data were collected from 15~24 cells in a total of 6~8 coverslips, where 2 coverslips was obtained from each of 3~4 independent litters of mice. Recording were made from 2~5 cells per coverslip. The number of independent litters, N, and the number of cells, n, are indicated in the bar graph as N/n. Results from one-way ANOVA analysis are provided in Supplementary Table 7.

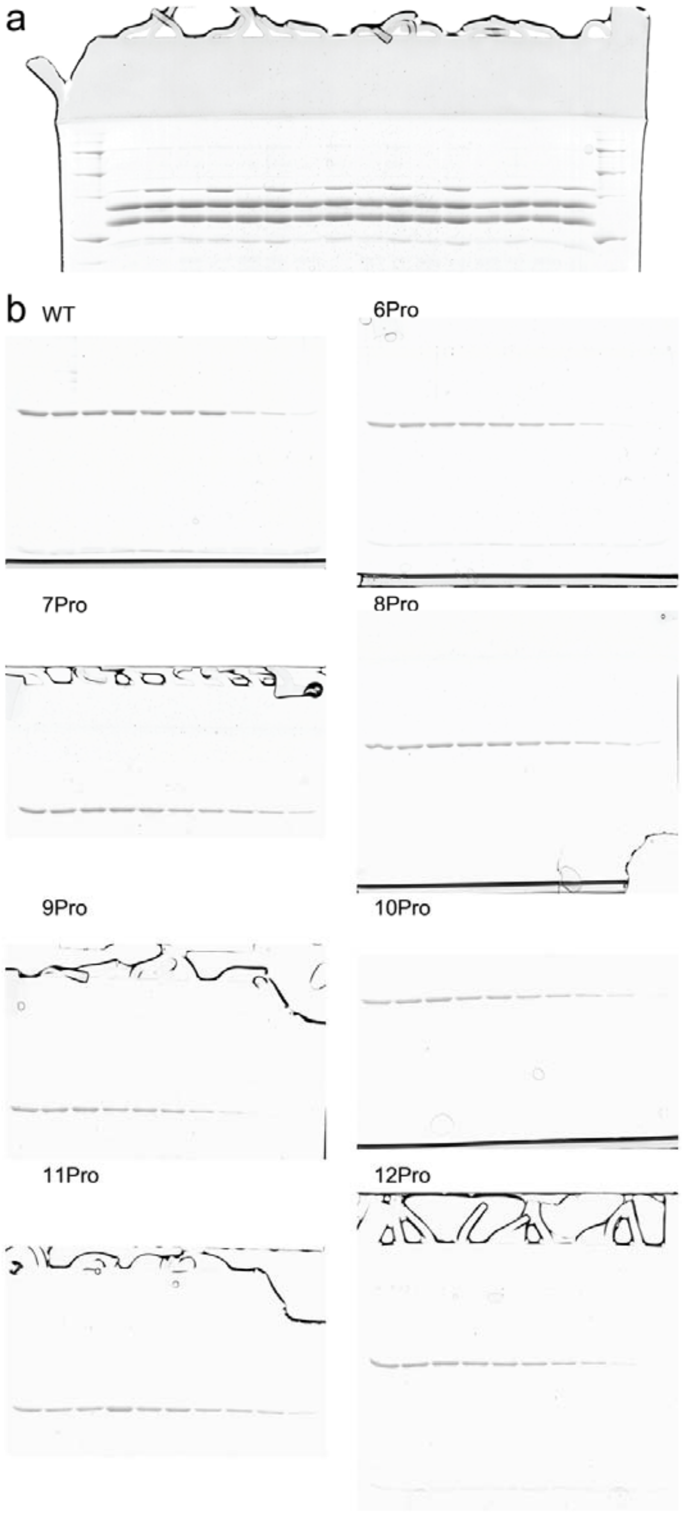

Supplementary Fig. 5. Uncropped images of entire gels associated with Supplementary Fig. 1a (**a**) and Supplementary Fig. 2a (**b**).

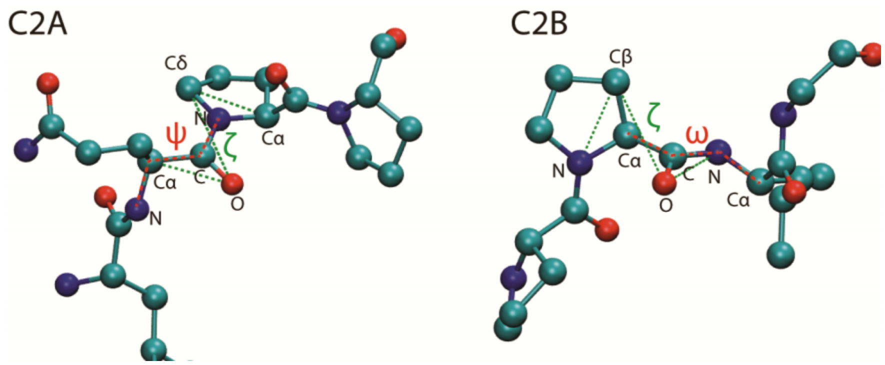

Supplementary Figure 6. Dihedral angles used to characterize the rotational flexibility of the poly-proline rod, with respect to the C2-domains, at the linkage point. These dihedral angles are used as collective variables in the metadynamics simulations.

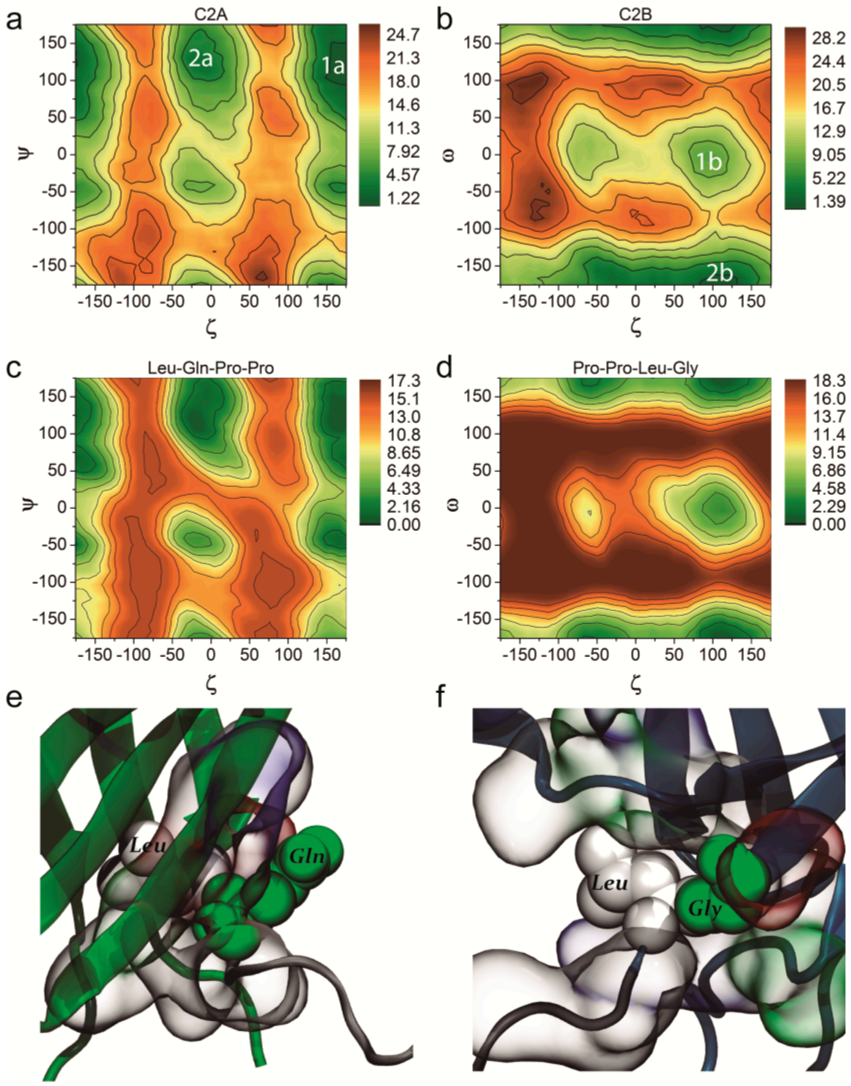

Supplementary Figure 7. Metadynamics simulations that characterize the relative orientation of the poly-proline segment and individual C2-domains. (**a**) Free energy surface for C2A-9Pro. The collective variables are (see Supplementary Fig. 6: ζ, the pseudo dihedral angle Gln:CA-Gln:O-Pro:CD-Pro:CA, and ψ, the dihedral angle Gln:N-Gln:CA-Gln:C-Pro:N. The low-free-energy basins are indicated as **1a** and **2a**. (**b**) Free energy surface for C2B-9Pro. The collective variables are (see Supplementary Fig. 6: ζ, the pseudo dihedral angle Pro:N-Pro:CB-Pro:O-Leu:N, and ω, the dihedral angle Pro:CA-Pro:C-Leu:N-Leu:CA. The low-free-energy basins are labeled as **1b** and **2b**. (**c**) Free energy surface for a tetra-peptide model for the linkage between C2A and a poly-proline segment: Leu-Gln-Pro-Pro. (**d**) Free energy surface for a tetra-peptide model for the linkage between C2B and a poly-proline segment: Leu-Gln-Pro-Pro. (**e**) A snapshot from the metadynamics simulation illustrates the steric interaction between Leu and Gln residues at the linkage between C2A and the poly-proline segment that controls the relatively fixed orientation of the poly-proline relative to the C2A domain. (**f**) Same as in panel e, but focused on the linkage between C2B and the poly-proline segment.

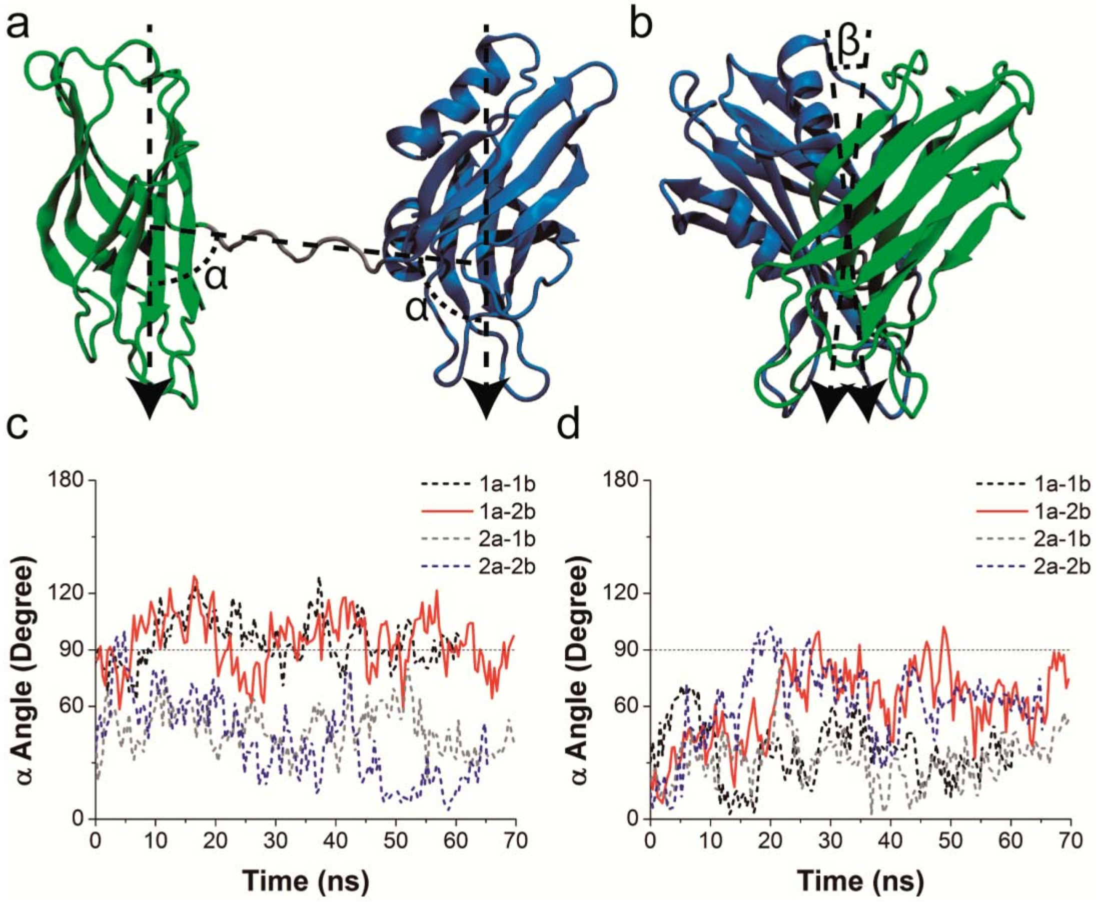

Supplementary Figure 8. Orientation of the Ca^{2+} binding loops in the C2-domains. (**a**) Schematic illustration of the α angles that represent the orientations of the Ca^{2+} binding loops with respect to the *n*Pro linker. C2A is green, C2B is blue, and the poly-proline linker is gray. (**b**) Schematic illustration of the β angles that represent the relative orientation of the Ca^{2+} binding loops in the two C2-domains. The arrows illustrate the orientations of the Ca^{2+} binding loops. (**c**) The C2A α angles in the four C2A-9Pro-C2B models (see text and Supplementary Table 8 during MD simulations. (**d**) The C2B α angles in the four C2A-9Pro-C2B models during MD simulations.

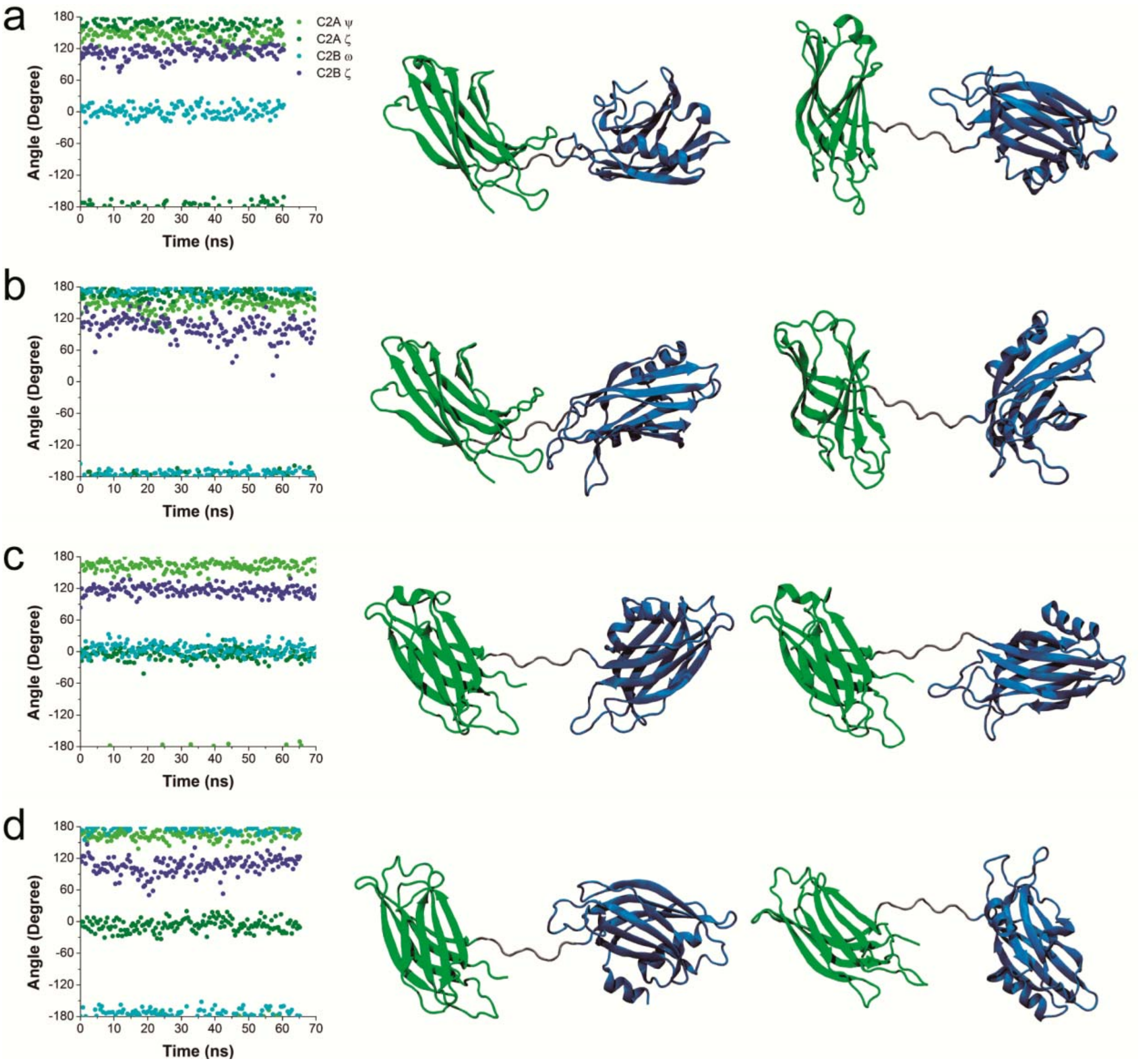

Supplementary Figure 9. Properties of the four C2A-9Pro-C2B models during MD simulations. The four rows summarize results for the (**a**) **1a-1b**, (**b**) **1a-2b**, (**c**) **2a-1b** and (**d**) **2a-2b** C2A-9Pro-C2B models, respectively. For each model, the left panel shows the time dependence of the collective variables used in the metadynamics simulations for C2A-9Pro and C2B-9Pro; the right panel illustrates the starting and final structures of each C2A-9Pro-C2B simulation. C2A is green, C2B is blue, and the poly-proline linker is gray.

Supplementary Table 1: Time-to-peak of evoked EPSCs recorded from WT, syt KO, and syt KO neurons expressing WT or linker mutant forms of syt

		time-to-peak (ms)	n	*p*-value vs WT	*p*-value vs syt KO
WT		19.2 ± 1.0	25		
syt KO		41.3 ± 7.3	21	0.014	
	WT	18.5 ± 0.6	33	0.551	0.005
	6Pro	20.7 ± 0.6	31	0.234	0.01
	7Pro	20.0 ± 1.0	32	0.593	0.009
	8Pro	19.9 ± 1.1	32	0.655	0.008
	9Pro	19.5 ± 0.7	31	0.855	0.007
	10Pro	18.9 ± 1.0	32	0.826	0.006
	11Pro	18.7 ± 1.1	32	0.743	0.006
	12Pro	17.2 ± 0.7	27	0.113	0.003

Note: Time-to-peak is defined as the time between the start of stimulation and the peak current. Data are represented as the mean ± SEM. *p*-values were determined using the Student’s t-test.

Supplementary Table 2. Sequences of the primers used in this study.

Primers used to generate proline linker mutants of syt	
6Pro Sense	GAGTGGCGTGATCTCCAGCCGCCGCCGCCGCCGCTGGGTGACATCTGCTTCTCC
6Pro Antisense	GGAGAAGCAGATGTCACCCAGCGGCGGCGGCGGCGGCTGGAGATCACGCCACTC
7Pro Sense	GAGTGGCGTGATCTCCAGCCGCCGCCGCCGCCGCCGCTGGGTGACATCTGCTTC
7Pro Antisense	GAAGCAGATGTCACCCAGCGGCGGCGGCGGCGGCGGCTGGAGATCACGCCACTC
8Pro Sense	GGCGCGATCTCCAGCCGCCGCCGCCGCCGCCGCCGCTGGGTGACATCTGC
8Pro Antisense	GCAGATGTCACCCAGCGGCGGCGGCGGCGGCGGCGGCTGGAGATCGCGCC
9Pro Sense	GCGATCTCCAGCCGCCGCCGCCGCCGCCGCCGCCGCTGGGTGACAT
9Pro Antisense	ATGTCACCCAGCGGCGGCGGCGGCGGCGGCGGCGGCTGGAGATCGC
10Pro Sense	GGCGCGATCTCCAGCCGCCGCCGCCGCCGCCGCCGCCGCCGCTGGGTGACATCTGC
10Pro Antisense	GCAGATGTCACCCAGCGGCGGCGGCGGCGGCGGCGGCGGCGGCTGGAGATCGCGCC
11Pro Sense	CGCGATCTCCAGCCGCCGCCGCCGCCGCCGCCGCCGCCGCCGCTGGGTGACATCTG
11Pro Antisense	CAGATGTCACCCAGCGGCGGCGGCGGCGGCGGCGGCGGCGGCGGCTGGAGATCGCG
12Pro Sense	GCGATCTCCAGCCGCCGCCGCCGCCGCCGCCGCCGCCGCCGCCGCTGGGTGACATC
12Pro Antisense	GATGTCACCCAGCGGCGGCGGCGGCGGCGGCGGCGGCGGCGGCGGCTGGAGATCGC
Primers used to generate point mutant forms of syt	
C277A Sense	GAGAAACTGGGTGACATCGCCTTCTCCCTCCGCTACGT
C277A Antisense	ACGTAGCGGAGGGAGAAGGCGATGTCACCCAGTTTCTC
F234C Sense	GTGTATGACTTTGATCGCTGTTCCAAGCACGACATCATC
F234C Antisense	GATGATGTCGTGCTTGGAACAGCGATCAAAGTCATACAC
I367C Sense	GTTTTGGACTATGACAAGTGTGGCAAGAACGACGCCATC
I367C Antisense	GATGGCGTCGTTCTTGCCACACTTGTCATAGTCCAAAAC

Supplementary Table 3: sine wave functions parameters

Equation: $y = a + b \times sin\left(\frac{x - c}{s} \times 2\pi\right)$

Figure	Adjusted R^2	a	b	c
Fig. 2c	0.67	46.8 ± 0.80	4.12 ± 1.10	-1.04 ± 0.13
Fig. 3d	0.71	6.04 ± 0.42	-2.31 ± 0.56	0.73 ± 0.13
Fig. 3e	0.73	5.04 ± 0.30	1.71 ± 0.40	-0.59 ± 0.12
Fig. 4b	0.74	255 ± 15	-89.9 ± 20.4	0.76 ± 0.12
Fig. 5b	0.81	2.77 ± 0.45	2.19 ± 0.57	-0.75 ± 0.15
Fig. 6b	0.78	0.11 ± 0.009	0.064 ± 0.01	-0.81 ± 0.1
Supplementary Fig. 1c	0.35	0.33 ± 0.02	-0.05 ± 0.02	1.16 ± 0.21
Supplementary Fig. 2b	0.95	0.13 ± 0.003	0.047 ± 0.005	-2.04 ± 0.05

Supplementary Table 4: ANOVA results for data in Fig. 4b

ANOVA summary
F = 15.45
p-value < 0.0001
The differences among mea are statistically significant at 0.001 level.

	WT	Syt 1 KO	KO + WT	KO + 6Pro	KO + 7Pro	KO + 8Pro	KO + 9Pro	KO + 10Pro	KO + 11Pro	KO + 12Pro
n	25	21	33	31	32	32	31	32	32	27

Pairwise Tukey's multiple comparisons test:

Adjusted p-values	WT	Syt1 KO	KO + WT	KO + 6Pro	KO + 7Pro	KO + 8Pro	KO + 9Pro	KO + 10Pro	KO + 11Pro	KO + 12 Pro
Syt 1 KO	< 0.0001									
KO + WT	> 0.9999	< 0.0001								
KO + 6Pro	0.5036	< 0.0001	0.7478							
KO + 7Pro	< 0.0001	0.0465	< 0.0001	0.0196						
KO + 8Pro	< 0.0001	0.1107	< 0.0001	0.0058	> 0.9999					
KO + 9Pro	0.746	< 0.0001	0.9269	> 0.9999	0.0052	0.0013				
KO + 10Pro	0.0012	0.0008	0.0026	0.4424	0.9584	0.833	0.2214			
KO + 11Pro	0.002	0.0005	0.0045	0.5411	0.9209	0.7524	0.2945	> 0.9999		
KO + 12Pro	> 0.9999	< 0.0001	> 0.9999	0.5572	< 0.0001	< 0.0001	0.7955	0.0013	0.0023	

Significance	WT	Syt1 KO	KO + WT	KO + 6Pro	KO + 7Pro	KO + 8Pro	KO + 9Pro	KO + 10Pro	KO + 11Pro	KO + 12 Pro
Syt 1 KO	***									
KO + WT		***								
KO + 6Pro		***								
KO + 7Pro	***	*	***	*						
KO + 8Pro	***		***	**						
KO + 9Pro		***			**	**				
KO + 10Pro	**	***	**							
KO + 11Pro	**	***	**							
KO + 12Pro		***			***	***		**	**	

* p < 0.05, ** p < 0.01, and *** p < 0.001

Supplementary Table 5: ANOVA results for data in Fig. 5b

ANOVA summary
F = 9.9
p-value < 0.0001
The differences among means are statistically significant at 0.001 level.

	WT	Syt 1 KO	KO + WT	KO + 6Pro	KO + 7Pro	KO + 8Pro	KO + 9Pro
n	25	28	24	21	24	21	24

Pairwise Tukey's multiple comparisons test:

Adjusted p-values	WT						
Syt 1 KO	0.0035	Syt1 KO					
KO + WT	0.9998	0.0151	KO + WT				
KO + 6Pro	< 0.0001	0.695	0.0001	KO + 6Pro			
KO + 7Pro	> 0.9999	0.0026	0.9988	< 0.0001	KO + 7Pro		
KO + 8Pro	> 0.9999	0.004	0.9988	< 0.0001	> 0.9999	KO + 8Pro	
KO + 9Pro	0.0078	> 0.9999	0.0289	0.6748	0.0058	0.0083	KO + 9Pro

Significance	WT						
Syt 1 KO	**	Syt 1 KO					
KO + WT		*	KO + WT				
KO + 6Pro	***		***	KO + 6Pro			
KO + 7Pro		**		***	KO + 7Pro		
KO + 8Pro		**		***		KO + 8Pro	
KO + 9Pro	**		*		**	**	KO + 9Pro

* p < 0.05, ** p < 0.01, and *** p < 0.001

Supplementary Table 6: ANOVA results for data in Fig. 5c

ANOVA summary
F = 0.4793
p-value = 0.823
The differences among means are not statistically significant at 0.05 level.

	WT	Syt 1 KO	KO + WT	KO + 6Pro	KO + 7Pro	KO + 8Pro	KO + 9Pro
n	25	28	24	21	24	21	24

Pairwise Tukey's multiple comparisons test:

Adjusted p-values	WT						
Syt 1 KO	0.9996	Syt1 KO					
KO + WT	0.9997	> 0.9999	KO + WT				
KO + 6Pro	0.9856	0.8929	0.908	KO + 6Pro			
KO + 7Pro	0.9983	0.9633	0.9696	> 0.9999	KO + 7Pro		
KO + 8Pro	0.9991	0.9758	0.9798	> 0.9999	> 0.9999	KO + 8Pro	
KO + 9Pro	0.9973	0.9537	0.9614	> 0.9999	> 0.9999	> 0.9999	KO + 9Pro

Significance	WT						
Syt 1 KO		Syt 1 KO					
KO + WT			KO + WT				
KO + 6Pro				KO + 6Pro			
KO + 7Pro					KO + 7Pro		
KO + 8Pro						KO + 8Pro	
KO + 9Pro							KO + 9Pro

* p < 0.05, ** p < 0.01, and *** p < 0.001

Supplementary Table 7 ANOVA results for data in Supplementary Fig. 4a

ANOVA summary

F = 4.255

p-value < 0.0001

The differences among mea are statistically significant at 0.001 level.

	WT	Syt 1 KO	KO + WT	KO + 6Pro	KO + 7Pro	KO + 8Pro	KO + 9Pro	KO + 10Pro	KO + 11Pro	KO + 12Pro
n	24	22	23	19	15	20	18	19	17	20

Pairwise Tukey's multiple comparisons test:

Adjusted p-values	WT	Syt1 KO	KO + WT	KO + 6Pro	KO + 7Pro	KO + 8Pro	KO + 9Pro	KO + 10Pro	KO + 11Pro	KO + 12 Pro
Syt 1 KO	0.0032									
KO + WT	> 0.6957	< 0.0001								
KO + 6Pro	0.9997	0.0008	0.9715							
KO + 7Pro	> 0.9999	0.0132	0.8347	> 0.9999						
KO + 8Pro	> 0.9999	0.0022	0.8829	> 0.9999	> 0.9999					
KO + 9Pro	> 0.9999	0.0066	0.807	> 0.9999	> 0.9999	> 0.9999				
KO + 10Pro	0.9984	0.0004	0.9889	> 0.9999	0.9995	> 0.9999	0.9995			
KO + 11Pro	0.8022	< 0.0001	> 0.9999	0.9854	0.892	0.9314	0.8764	0.9948		
KO + 12Pro	> 0.9999	0.0017	0.9052	> 0.9999	> 0.9999	> 0.9999	> 0.9999	>0.9999	0.9459	

Significance	WT	Syt1 KO	KO + WT	KO + 6Pro	KO + 7Pro	KO + 8Pro	KO + 9Pro	KO + 10Pro	KO + 11Pro	KO + 12 Pro
Syt 1 KO	**									
KO + WT		***								
KO + 6Pro		***								
KO + 7Pro		*								
KO + 8Pro		**								
KO + 9Pro		**								
KO + 10Pro		***								
KO + 11Pro		***								
KO + 12Pro		**								

* p < 0.05, ** p < 0.01, and *** p < 0.001

Supplementary Table 8 Relative free energy (ΔG, in kcal/mol) of dominant conformers based on metadynamics simulations for C2A/B-9Pro systems. See Supplementary Fig. 7for the labels of the conformers.

C2A/B-9Pro basins	C2A-1a	C2A-2a	C2B-1b	C2B-2b
Computed ΔG	0.0	2.2	7.1	0.0
C2A-9Pro-C2B models	1a-1b	1a-2b	2a-1b	2a-2b
Estimated ΔG	7.1	0.0	9.3	2.2

Supplementary Notes 1

To determine the relative orientation of the tandem C2-domains of syt, when connected by a poly-proline rod, and to determine whether these domains are constrained, two types of molecular simulations were carried out. First, metadynamics simulations were used to compute the free energy surfaces for the rotation of the poly-proline rod with respect to each individual C2-domain. This information was then used to construct models for the tandem C2-domains connected by poly-proline linkers of nine, ten and eleven residues, and to compute the relative orientation of the tandem domains.

Metadynamics simulations for individual C2-domains

As shown in Supplementary Fig. 7, the (ζ,ψ) free energy map for C2A-9Pro has two dominant basins with ζ~0 (basin **2a**) and 180° (basin **1a**); they are fairly close in free energy (~2 kcal/mol) but are separated by sufficiently high (~15.3 kcal/mol) barriers. These conformers are more stable, compared to the basins with ψ~-50°, due to the hydrophobic packing of the connecting Leu residue against the nearby Gln (Supplementary Fig. 7e; in fact, the free energy map is qualitatively very similar to that computed for the isolated tetra-peptide that corresponds to the C2A-9Pro linkage (Leu-Gln-Pro-Pro, Supplementary Fig. 7c). Similarly, as shown in Supplementary Fig. 7b, the (ζ,ω) free energy map for C2B-9Pro is also highly similar to that for the isolated tetra-peptide that corresponds to the C2B-9Pro linkage (Pro-Pro-Leu-Gly, Supplementary Fig. 7d). The dominant basins (**1b** and **2b**) have ζ~120°, to avoid the steric collision between Leu side chain and the proline rings (Supplementary Fig. 7f); the free energy difference between the two basins is rather large (~7 kcal/mol) since ω~180° is the preferred conformation in the peptide.

Conformer 1a-2b represents the energetically most stable C2A-9Pro-C2B model

The free energy maps indicate that for both C2-domain-9Pro linkages, the relative orientation of the poly-proline segment and the C2-domain is relatively rigid. Therefore, it is possible to build C2A-9Pro-C2B models by combining the low free-energy conformers for C2A-9Pro and C2B-9Pro; taking two dominant conformers for each (1a/2a and 1b/2b), we arrived at four possible models for C2A-9Pro-C2B. These four models were each simulated for ~70 ns using NPT molecular dynamics (Supplementary Fig. 9).

With the assumption that there is minimal interaction between the two C2-domains when separated by a poly-proline rod, the relative stabilities of the four models can be estimated by combining the free energies of different C2-domain-linker conformers (see Supplementary Table 8. The results indicate that only two C2A-C2B conformations, **1a-2b** (Supplementary Fig. 7b) and **2a-2b** (Supplementary Fig. 7d) are relevant at RT, with the former being the dominant configuration with a population of about 98%. We note that the approximation of minimal domain-domain interaction in C2A-9Pro-C2B appears a valid one since the two domains indeed remain far apart in all four relaxed models (see Supplementary Fig. 9); although the minimal domain-domain distances (data not shown) may reach ~5-10 Å during some segments of the trajectories, persistent inter-domain contacts were not observed. Moreover, the internal

structures of the two domains in the C2A-9Pro-C2B simulations remain very similar to those in the isolated C2-domain-9Pro simulations, again supporting the assumption of minimal domain interactions. Finally, the collective variables chosen in the metadynamics simulations remain close to their expected values during all C2A-9Pro-C2B simulations (Supplementary Fig. 9 first column), confirming that these dihedral angles remain the key variables for characterizing the relative poly-proline/C2 orientations; one C2-domain is not strongly perturbed by the presence of the other C2-domain. The fact that these dihedral angles remain largely constant during the simulations again highlight the relative rigidity of the poly-proline/C2 connection; the C2-domains, however, have sufficient time during the simulation to equilibrate their local interactions as indicated by the changes in the orientation of the Ca^{2+}-binding loops relative to the poly-pro linker (see beginning and final structures shown in Supplementary Fig. 9 and the time dependence of the alpha angles in Supplementary Fig. 8). Therefore, although a more precise estimate of the populations of these conformers could be obtained with metadynamics simulations for the C2A-9Pro-C2B system in the presence of both calcium ions and multi-component lipid membrane, which would be highly demanding computationally (in addition to the concern of using a non-polarizable force field for calcium-protein interactions[1]), the computational results presented herein provide adequate support for **1a-2b** being the dominant conformation for the tandem C2-domains connected with a poly-proline rod, especially viewed together with results from penetration assay in this study and the NMR data from Ref. [2]. In subsequent MD simulations for C2A-*n*Pro-C2B as *n* is varied from 9 to 11, only the **1a-2b** conformer is considered. The observation that the 2a-2b conformation is estimated to be only ~2 kcal/mol higher in free energy is consistent with the experimental observation that an alternative C2A-C2B orientation is likely to play a different physiological role during exocytosis).

Supplementary references

1. Li, H., *et al.* Representation of Ion–Protein Interactions Using the Drude Polarizable Force-Field. *J. Phys. Chem. B* **119**, 9401-9416 (2015).

2. Seven, A.B., Brewer, K.D., Shi, L., Jiang, Q.X. & Rizo, J. Prevalent mechanism of membrane bridging by synaptotagmin-1. *Proc. Natl. Acad. Sci. USA* **110**, E3243-3252 (2013).
